# Sociodemographic and Clinical Correlates Associated with the Frequent Service Users in an Italian Psychiatric Emergency Department

**DOI:** 10.3390/diagnostics13030430

**Published:** 2023-01-25

**Authors:** Andrea Aguglia, Giovanni Pietro Corsini, Alessandra Costanza, Andrea Berti, Edoardo Bruno, Andrea Escelsior, James Sanvi, Alice Trabucco, Eleonora Vai, Andrea Amerio, Gianluca Serafini, Mario Amore

**Affiliations:** 1 Department of Neuroscience, Rehabilitation, Ophthalmology, Genetics, Maternal and Child Health, Section of Psychiatry, University of Genoa, 16132 Genoa, Italy; 2 Istituto di Ricovero e Cura a Carattere Scientifico (IRCCS), Ospedale Policlinico San Martino, 16132 Genoa, Italy; 3 Department of Psychiatry, Faculty of Medicine, University of Geneva (UNIGE), 1211 Geneva, Switzerland; 4 Department of Psychiatry, Adult Psychiatry Service (SPA), University Hospitals of Geneva (HUG), 1211 Geneva, Switzerland

**Keywords:** frequent service users, substances, personality disorders, mood disorders, emergency psychiatry, cocaine, cannabinoid

## Abstract

Background: The aim of the present study is to identify the main sociodemographic and clinical correlates associated with frequent service users (FSUs) in an Italian psychiatric emergency department. Methods: This study is an observational and prospective clinical investigation. All subjects (N = 549) consecutively admitted to the Psychiatric Inpatient Unit of the IRCCS Ospedale Policlinico San Martino ((Genoa, Italy) were recruited over a period of 18 months. Results: On average, FSUs were more likely to be single (75.0% vs. 64.0, *p* = 0.001), younger (38.79 years ± 14.68 vs. 45.94 years ± 16.94, *p* = 0.028), with an earlier onset (20.15 years ± 7.22 vs. 29.33 years ± 15.96, *p* < 0.001), and longer length of hospitalisation (13.65 days ± 12.40 vs. 9.89 ± 10.15, *p* = 0.006) compared to non-FSUs. While bipolar disorder was the most common primary diagnosis in both FSUs and non-FSUs, cluster B personality disorder was particularly elevated in FSUs (30.3% vs. 10.4%, *p* < 0.001). Furthermore, FSUs were more prone to substance use disorder (63.6% vs. 40.0%, *p* < 0.001), particularly cannabis (45.5% vs. 15.3%, *p* < 0.001), cocaine (33.3% vs. 10.4%, *p* < 0.001), and heroin (19.7% vs. 5.8%, *p* < 0.001), and were more likely to have non-suicidal self-injuries (21.2% vs. 6.8%, *p* < 0.001). FSUs were significantly more likely to be discharged against medical advice (18.2% vs. 5.6%, *p* < 0.001) or to have at least one escape attempt from the psychiatric ward (12.1% vs. 0.8%, *p* < 0.001). Conclusions: Specific clinical and social profiles of patients who repeatedly utilised the services of a psychiatric emergency department have been identified. Our findings can be used to develop suitable structures to support and reintegrate FSUs into society and work life.

## 1. Introduction

The reorganisation of the mental health system in Italy during the last two decades of the 20th century led to the closing of several psychiatric hospitals. Consequently, community-based services were established for acute, sub-acute, and residential care settings in combination with a significant reduction of acute inpatient psychiatric beds, which continues to affect overall patient access to care [1]. In fact, psychiatric admissions in Italy are currently based on the individual’s clinical and psychopathological conditions with psychiatric admissions regulated by a mental health law (Law 180/78), that allows for both voluntary and involuntary psychiatric hospitalisations.

This important legislative change prescribes a more moderate therapeutic approach, based on outpatient services, and encourages the reintegration of patients into their social environment whenever possible. However, individuals with acute clinical conditions are hospitalised in psychiatric emergency departments where they typically remain for a brief period of time before being discharged, often prematurely, due to the limited availability of beds. These shorter hospitalisations alone already lead to an increased risk of readmission and do not decrease the total number of treated inpatients [2,3]. Overall, up to 13% of psychiatric patients require rehospitalisation over relatively short time spans [1,4,5,6,7]. This phenomenon has been termed “revolving door” by some authors or “frequent service users (FSUs)” by others. The definition of FSUs varies in the literature but is usually defined by the number of recurrent admissions within a certain time interval [5,8,9,10,11,12,13,14,15]. Overall, the most widely adopted definition of FSUs refers to patients with three or more hospitalisations during a 12-month period. Researchers and politicians increasingly pushed for this topic to become a public health issue due to the growing direct and indirect costs and a worsening cost/benefit ratio [16,17,18]. This has resulted in an international trend of deinstitutionalization as inpatient care is the most costly component of mental health systems; hence, minimising both the duration of admissions and the number of readmissions have become clinical and strategic priorities worldwide and, although controversial among health care professionals, are used by decision markers as quality indicators of mental health care. This has led to improper and incorrect bed occupancy and has negatively impacted the workload and stress level of healthcare personnel who, based on these new performance indicators, have been accused of repetitive therapeutic failure [8,19]. As a result, most of the current research has been focused on trying to explain readmission in terms of sociodemographic and clinical factors and the characteristics of individual mental health services, while very few studies examined readmissions from the FSU’s perspective [20]. Generally, psychiatric inpatients have the greatest risk of adverse outcomes (e.g., rehospitalisation, relapse, suicide) right after discharge. Mutscher et al. [21] reviewed studies on the transition process from psychiatric hospitalisation to the community. They identified several themes necessary for a successful transition, including safety, supported autonomy, and the opportunity to engage in a number of reintegration activities, while poverty, interpersonal difficulties, and stigma represented the main barriers. One of the few studies, focussing on the FSUs perspective of rehospitalisations, was presented by Duhig et al. [17] who concluded within an Australian context that hospitalisation often represented a relief/safe haven from individual coping mechanisms, stress, and an inhospitable world, and was either the default coping mechanism or the last resort after unsuccessful and outright counterproductive attempts to self-manage stressful circumstances. With regard to the scientific approach, they suggested to view the FSUs phenomenon as a process rather than a succession of individual events to facilitate a more recovery-oriented approach. Some of these findings, particularly regarding the relief/safe haven perceptions, were also found by Ådnanes et al. [22] in study spanning six European countries. Further, they found that rehospitalisations were less traumatising that the first hospitalisation, particularly if the latter was involuntary. Many FSUs regarded rehospitalisations either as necessary, e.g., during a time of crisis, or as outright inevitable because they were unable to obtain adequate support outside the hospital environment, sometimes fully aware that they had evolved a condition of institutionalisation, although the rehospitalisations were not necessarily perceived as resulting in lasting progress or recovery.

Identifying the variables that influence FSUs is of primary importance to understand and reduce/prevent this phenomenon, which is often accompanied with a worse prognosis and negative outcomes in terms of course, well-being, quality of life, and functioning [6,8,15]. Indeed, the accurate identification of risk factors for rehospitalisation is crucial for improving (i) the discharge planning, (ii) the knowledge of the course of chronic psychiatric disorders and treatment effects, and (iii) the effective allocation of public resources and organising public health systems. Further, a better understanding of risk factors for rehospitalisation may potentially suggest modifiable neural mechanisms common to FSUs [23].

Several studies found that FSUs were more frequently male, younger, single, unemployed, and often exhibiting several psychiatric conditions such as mood disorders (particularly patients with bipolar disorder), substance use disorders, personality disorders, lifetime non-suicidal self-injuries and suicide attempts (SAs), psychotic disorders, and anorexia nervosa [6,18,24,25,26,27,28,29]. FSUs with cluster B personality, bipolar disorder, substance disorder, lifetime non-suicidal self-injuries or SAs have been found to be impulsive with impulsivity-related endophenotypes (aggression and trait anger), which may explain the repeated visits to psychiatric emergency departments [30,31]. Higher FSUs rates were also found in individuals with a history of electroconvulsive therapy, multiple psychopharmacological treatments, including clozapine use, and lack of compliance [29]. The latter, a lack of adherence to treatment, emerged as particularly important [10,15,32,33]. Furthermore, interindividual differences in the enzyme activity of cytochrome P450 2D6 could be responsible for adverse drug reactions and consequently for significant therapeutic failures, which could ultimately contribute to frequent rehospitalisations and FSU status. This is particularly true when it occurs with different psychiatric disorders, especially mood disorders, characterised by a chronic course interspersed with episodic affective recurrences [32,33]. Moreover, there is also an interplay between individual and systemic factors, such as homelessness and unemployment (both reinforced by manifestations of stigmatisation that increase with each rehospitalisation), poor support networks, challenging social environments, disability, low socioeconomic status, or individuals deprived of citizenship rights which prevents them from accessing education and pursuing adequate employment opportunities, while outpatient facilities may simply be inadequate or overwhelmed [3,6,20].

This study aims to offer novel data that could help improve identification of at-risk patients, shape possible treatment strategies, and facilitate a more efficient distribution of health care resources.

## 2. Materials and Methods

### 2.1. Sample

This study is an observational and prospective clinical investigation, designed to identify the main sociodemographic and clinical correlates associated with FSUs phenomenon in an Italian psychiatric emergency department. All patients consecutively admitted to the Psychiatric Inpatient Unit of the Istituto di Ricovero e Cura a Carattere Scientifico (IRCCS) Ospedale Policlinico San Martino (Genoa, Italy), over a period of 18 months (from 1 January 2019 to 30 June 2020), were recruited (N = 549). In accordance with previous studies, FSUs were defined by patients with at least three hospitalisations within the required 12-month follow-up period following the index episode [1,5,8,26]. All patients were provided with an adequate and detailed explanation of the study design and written informed consent was obtained prior to their recruitment, in accordance with the current version of the Declaration of Helsinki [34]. The study design was approved by the local Ethics Review Board.

### 2.2. Clinical Assessment

Basic sociodemographic and clinical characteristics were recorded during a semi-structured interview which covered the following areas: sociodemographic variables (age, gender, marital and occupational status, education level, residential and migrant status); clinical parameters (psychiatric diagnosis at discharge, age at onset, duration of illness, non-suicidal self-injuries, and suicidal behaviour (SB) (i.e., lifetime SAs)); length of current hospitalisation (days), calculated as elapsed time between admission and discharge; type of admission as either voluntary or involuntary; need of physical restraint; presence of psychiatric and/or medical comorbidity; presence of at least one illicit substance (e.g., alcohol, cannabis, cocaine, amphetamines, heroin, and novel substances); and pharmacological treatment (i.e., number of medications, oral or long-acting injection antipsychotics, antidepressants, mood stabilisers, benzodiazepines, and others). Finally, other variables (family history of psychiatric disorders, living situation, voluntary discharge against medical advice, escape attempt from the psychiatric ward) were also investigated.

All patients were diagnosed according to the Diagnostic and Statistical Manual for Mental Disorders (DSM), fifth edition [35], and the consensus of at least three providers. The diagnoses at discharge were divided into the following subgroups: schizophrenia-related disorders (referred to hereinafter as schizophrenia), bipolar and related disorders, depressive disorders, substance use disorders, personality disorders, and others (including the remaining psychiatric and non-psychiatric disorders such as social admission diagnosis). Recruited patients from our catchment area were assessed only if two psychiatrists considered their psychopathological conditions as clinically stable. If patients were affected by more than one psychiatric diagnosis, only the primary condition was recorded in line with the pharmacological treatment prescribed by a senior psychiatrist (with more than ten years of clinical experience in the field) [36,37].

### 2.3. Statistical Analysis

Sociodemographic and clinical characteristics are presented either as mean ± standard deviation (SD) or as absolute counts and percentages for continuous and categorical variables, respectively. They were assessed for normal distributions using the Kolmogorov–Smirnov test. The sample was divided into two subgroups according to their FSUs status (see definition above). Pearson’s chi-squared test with Yates’ correction, and a t-test for independent samples, were performed for statistical comparisons.

We used a logistic regression analysis using as dependent variable “FSUs group” and including sequentially sociodemographic, clinical and other characteristics potentially associated with FSUs. So, we would create a set of theoretically driven logistic models which could be compared to see whether there is an incremental benefit of the addition of new variables (the first model included only sociodemographic variables, the second model included also clinical variables and the third model included other characteristics investigated).

All statistical analyses were performed using Statistical Package for Social Sciences (SPSS Inc., Chicago, IL, USA; v. 24.0 for Windows), with the statistical significance threshold set to *p* < 0.05 (two-tailed).

## 3. Results

Overall, from the total sample of 549 subjects included in the study, 66 (12.0%) met our definition of FSUs. The mean ± SD age of the total sample was 45.87 ± 16.42 years with 314 (57.2%) males, and about one in four (24.0%) employed at the time of admission. The most frequent primary diagnosis was bipolar disorder (N = 166, 30.3%) followed by schizophrenia (N = 155, 28.2%). The mean duration of illness was 17.58 ± 13.43 years. Just over one-sixth of the sample (N = 96, 17.5%) had at least one lifetime SA. All sociodemographic and clinical characteristics are summarised in Table 1 and Table 2.

On average, FSUs were single (75.8% vs. 64.0%; *p* = 0.028), younger (38.79 years ± 14.68 vs. 45.94 years ± 16.94; *p* = 0.001), with an earlier age at onset (20.15 years ± 7.22 vs. 29.33 years ± 15.96; *p* < 0.001), and a longer length of hospitalisation (13.65 days ± 12.40 vs. 9.89 days ± 10.15; *p* = 0.006) than non-FSUs. Furthermore, FSUs showed cluster B personality disorder as the primary psychiatric diagnosis (30.3% vs. 10.4%; *p* < 0.001) and were characterised by a higher prevalence of non-suicidal self-injuries (21.2% vs. 6.8%; *p* < 0.001) and lifetime SAs (1.76 ± 1.09 vs. 1.34 ± 0.64; *p* = 0.031). Finally, FSUs were more likely to be discharged to a public residence (30.3% vs. 17.8%; *p* = 0.019) or against medical advice (18.2% vs. 5.6%; *p* < 0.001), and to have at least one escape attempt from the psychiatric ward (12.1% vs. 0.8%; *p* < 0.001). All statistical comparisons are shown in Table 1 and Table 2.

Alcohol (40.9% vs. 27.3%; *p* = 0.023), cannabinoid (45.5% vs. 15.3%; *p* < 0.001), cocaine (33.3% vs. 10.4%; *p* < 0.001), amphetamines (4.5% vs. 1.4%; *p* = 0.047), and heroin (19.7% vs. 5.8%; *p* < 0.001), but not novel substances, were significantly related to FSUs (Table 3).

Regarding pharmacological treatment, FSUs were more likely to take a higher number of medications (3.74 ± 1.37 vs. 3.07 ± 1.47; *p* = 0.001): benzodiazepines (81.8% vs. 65.6%; *p* = 0.008), and other medications, including minor mood stabilizers (oxcarbazepine, pregabalin, gabapentin) or replacement treatment (such as methadone or buprenorphine) (31.8% vs. 14.1%; *p* < 0.001) compared to non-FSUs, without any other statistical differences. All findings are summarised in Table 4.

A logistic regression analysis was conducted step by step, including sequentially sociodemographic, clinical and other characteristics potentially associated with FSUs. Models 1 and 2 explained 4% and 22% of variance, respectively (R^2^ Nagelkerke = 0.041 and R^2^ Nagelkerke = 0.224) (see Supplementary Materials). Model 3 explained 38% of variance (R^2^ Nagelkerke = 0.384). A primary diagnosis of personality disorder, an earlier age at onset, a longer length of hospitalisation, the use of cannabinoid and, finally, having at least one escape attempt from the psychiatric ward and being discharged against medical advice were significantly associated with the FSU phenomenon (see Table 5).

## 4. Discussion

In our study, we reported the results of an observational and prospective clinical investigation designed to elucidate the main sociodemographic and clinical characteristics of FSUs in an Italian psychiatric emergency department, particularly focusing on correlates associated with repeat hospitalisations.

We attempted to establish a form of identikit of FSUs and the picture that has emerged from our analysis depicts typical FSUs as younger, single, with an earlier age at onset and a longer length of current hospitalisation, compared to non-FSUs. This is in good agreement with earlier studies [8,19,24,25,38]. However, unlike several of those studies, we did not observe any significant correlation with male gender. In further agreement with previous studies, we also found a higher prevalence of substance use [1,5,6,26,27], significantly elevated non-suicidal self-injuries, and slightly elevated SB (including SAs). Mood or psychotic disorders, which some authors found to be more prevalent [6,25,26,28,39], were either less frequent (depression) or equally frequent (schizophrenia) in our sample. Perhaps, the most striking difference to most previous studies is that we found a strong association between cluster B personality disorders and FSUs (OR = 2.141, CI 95% = 1.004–4.667; *p* = 0.050). It is well known that SB as well as mood or psychotic symptomatology are risk factors for rehospitalisation which is why clinicians should always screen and carefully investigate several clinical dimensions [40,41,42].

Our findings do not suggest that FSUs are misunderstood or neglected by mental health services as all FSUs in our study had early access to specialist services and a long history of medicalisation within the psychiatric circuit: they were patients with repeated psychiatric hospitalisations and had been for some time. Hence, prolonging the duration of hospitalisation does not necessarily seem to result in fewer rehospitalisations. However, by deciding to exclude all cases of rehospitalisation, that occurred before 8 weeks from the index event (see Methods), we likely underestimated the number of cases where therapeutic failure was associated with premature discharge.

FSUs were often integrated into a fixed therapeutic structure or community where the decision to discharge may not necessarily have been a purely clinical one but also dependent on other aspects, such as economic factors or patient fatigue, adversely affecting the relationship between the patient and centre and, thus, the effectiveness of the therapeutic strategy. In such cases, it may be advantageous for a patient to be placed in a different environment. If patients do not have a home to return to, they typically continue their therapeutic path in public health centres. If there are no places available, acute care wards often have to serve as temporary care centres until a more permanent home can be found for the patient, a situation that is far from ideal as it places additional and undue pressure on a system that already operates at capacity. In addition, the FSU phenomenon seems to be associated with a higher number of both escape attempts and voluntary discharges against medical advice. Thus, in many cases, a prolonged hospitalisation does not appear to strengthen and consolidate the therapeutic bond with the patient but produces a negative impact in terms of compliance and clinical outcome.

Regarding the clinical characteristics, bipolar disorder represents the most common primary diagnosis in both FSUs and non-FSUs, which is hardly surprising, considering that it is among the most common diagnoses in psychiatric emergency departments; moreover, patients with bipolar disorder are hospitalised more often during a manic or major depressive episode. The prevalence of cluster B personality disorder was significantly elevated in FSUs. This is not a novel finding, but rather reinforces what has been observed in other study populations, e.g., in a large cohort of Swiss patients, who were recurrent users of psychiatric emergency department [43]. The overall percentage of psychotic patients among FSUs was remarkably low. Evidently, psychotic patients capable of adhering to and complying with therapeutic requirements require less frequent rehospitalisation.

There was a remarkably high prevalence (63.6%) of substance use disorder in FSUs, particularly of sedatives (cannabinoid: 40.9% and alcohol: 45.5%), followed by a psychostimulant (cocaine: 33.3%). While these numbers are high, we should bear in mind that substance use is often found in psychiatric comorbidities or as a secondary diagnosis, especially in patients with bipolar disorder or borderline personality disorder. It has been reported that patients with comorbid substance use disorder have significantly increased rates of hospitalisations, bed days, and psychiatric emergency department contacts (*p* < 0.001) for the majority of the included substances, compared to patients without such disorders [27]. Sometimes, patients may seek out these substances for self-therapy purposes, as a deliberate form of self-medication, where the degree of awareness of the disease and adherence to psychopharmacological therapy is low or zero [44,45].

Similar to findings by Pasic [46] and Schmidt [47], our results indicate that the size and level of support from a patient’s family as well as their social and work-related networks affect the rehospitalisation rate. FSUs were less likely to live with family members or be married; furthermore, FSUs were more seldomly employed compared to non-FSUs, a condition that is likely to worsen in the near future due to the current economic crisis related to the Covid-19 pandemic [48]. The increased fragility or lack of relevant interpersonal relationships (also made more prominent by the Covid-19-related pandemic) is likely to have exacerbated the latter aspect concerning economic hardship, as the “interpersonal trust” was the only significant protective factor in previous periods of great economic uncertainty, e.g., in 2008 [49,50]. Hence, apart from individual factors, there are also a significant number of systemic factors at play, which may interact to reinforce one another (e.g., unemployment—homelessness—stigmatisation).

It is essential that, once the psychiatric pathology has been diagnosed, patients can obtain support from appropriate public structures and networks that can assist them in their reintegration into society and the workforce, preventing chronic istitutionalisation. A community-based programme, such as the “crisis home” model, first tested on a broad scale in Madison, Wisconsin, on psychiatric patients, including individuals affected by cluster B personality disorder, may be adopted as an approach to reduce FSUs phenomenon [51]. In this model, sponsor families take in patients undergoing a mental health crisis. It has been reported that violence was not a problem, partly because the patients were carefully screened, but also because they felt privileged to be accepted into someone’s home and they made an effort to behave with courtesy. This can be especially true for individuals with personality disorder, who tend to work better in a crisis home than in a hospital setting [52]. Results from a subsequent Swiss study confirmed the reduction of rehospitalisations and showed that, although the crisis home model was more cost effective than traditional hospitalisation, this did not reduce the quality of care, and patients suffering from severe mental illnesses appeared to benefit most from this programme [53]. This model belongs to the larger group of community-based treatment programmes, aimed at reducing the use of emergency departments and achieving the psychosocial rehabilitation and reintegration of patients through approaches that should be individualised, personalised and intensive [54].

Our study has several clinical implications, including the consideration of novel therapeutic approaches (both pharmacological and non-pharmacological) and post-discharge availability of multidisciplinary health personnel to provide a more personalised and structured therapeutic strategy to avoid rehospitalisation. Despite the importance of our findings, several limitations need to be considered. We divided the primary diagnoses into six main categories which may have affected the level of detail of our analysis, particularly for those patients in the “other diagnoses” category which was rather heterogeneous and non-specific. This study was limited to patients admitted to one Italian university hospital, which means that our findings may not be transferable to other health care systems or different geopolitical and socioeconomic regions. The findings from our regression model have provided a clinical profile of FSUs phenomenon, some of which with a large confidence interval: this could mean that several clinical dimensions investigated are closely influenced by each other, leading to a decrease of the alpha level of significance, generating more probability in finding the population parameter but at the same time creating more imprecision of the estimate. Furthermore, the results may suffer from a potential convenience sampling bias during the recruitment phase. Finally, in an effort to keep the study focused and concise, we decided not to delve into psychiatric comorbidities or secondary diagnoses, which might have yielded additional insight and information.

## 5. Conclusions

In this study, several statistically significant clinical and social correlates of patients who repeatedly utilised the psychiatric emergency department could be identified, including having a primary diagnosis of personality disorder, an early age at onset, a longer length of hospitalisation, and the use of cannabinoid. Other characteristics associated with FSUs were having at least one escape attempt from the psychiatric ward and being discharged against medical advice. Knowledge of the ‘typical’ FSUs does not provide unequivocal answers as to how we may limit and contain the FSU phenomenon, which is simply too ubiquitous and complex. However, patients included in our study neither lacked a correct diagnosis nor adequate recognition of their condition and they were not deprived of psychiatric institutionalisation or specialist support. The underlying causes for their rehospitalisations must, therefore, lie elsewhere, e.g., in the lack of rehabilitation programmes and adequate support projects. Psychiatric emergency departments are intended for acute patients and cannot provide long-term rehabilitative support. Our findings can be used to develop suitable structures to support and reintegrate FSUs into society and work life.

## Figures and Tables

**Table 1:** Sociodemographic characteristics of the entire sample and of the frequent service users (FSUs) and non-FSUs subgroups.

N (%) or Mean ± SD	Total Sample (N = 549)	FSUs (N = 66)	Non-FSUs (N = 483)	Chi^2^/ *t*-Test	*p*
Male Gender	314 (57.2)	36 (54.5)	278 (57.6)	0.215	0.643
Current age (years)	45.87 ± 16.42	38.79 ± 14.68	45.94 ± 16.94	3.269	0.001
Marital status				7.176	0.028
Single	359 (65.4)	50 (75.8)	309 (64.0)		
Married	86 (15.7)	3 (4.5)	83 (17.2)		
Separated/divorced/widowed	104 (18.9)	13 (19.7)	91 (18.8)		
Educational level (years)	10.63 ± 3.74	10.48 ± 3.34	10.76 ± 3.76	0.573	0.567
Occupational status (Yes)	132 (24.0)	13 (19.7)	119 (24.6)	0.776	0.378
Italian citizenship	491 (89.4)	60 (90.9)	431 (89.2)	0.172	0.678
Migrant status	69 (12.6)	6 (9.1)	63 (13.0)	0.826	0.364
Living situation				1.597	0.450
Alone	172 (31.3)	22 (33.3)	150 (31.1)		
With their family	308 (56.1)	33 (50.0)	275 (56.9)		
In public residence	69 (12.6)	11 (16.7)	58 (12.0)		
Discharge				7.883	0.019
Private home	324 (59.0)	38 (57.6)	286 (59.2)		
Transferred to other ward	119 (21.7)	8 (12.1)	111 (23.0)		
Public residence	106 (19.3)	20 (30.3)	86 (17.8)		
Discharge against medical advice	39 (7.1)	12 (18.2)	27 (5.6)	13.951	<0.001
Escape attempt from the psychiatric ward	12 (2.2)	8 (12.1)	4 (0.8)	34.636	<0.001

**Table 2:** Clinical characteristics of the entire sample and of the frequent service users (FSUs) and non-FSUs subgroups.

N (%) or Mean ± SD	Total Sample (N = 549)	FSUs (N = 66)	Non-FSUs (N = 483)	Chi^2^/ *t*-Test	*p*
Psychiatric diagnosis				26.061	<0.001
Schizophrenia and related disorders	155 (28.2)	13 (19.7)	142 (29.4)		
Bipolar and related disorder	166 (30.3)	20 (30.3)	146 (30.1)		
Depressive disorders	60 (10.9)	2 (3.0)	58 (12.0)		
Substance use disorders	44 (8.0)	7 (10.6)	37 (7.7)		
Personality disorders	70 (12.8)	20 (30.3)	50 (10.4)		
Other diagnoses	54 (9.8)	4 (6.1)	50 (10.4)		
Age at onset (years)	28.24 ± 15.48	20.15 ± 7.22	29.33 ± 15.96	4.606	<0.001
Duration of illness (years)	17.58 ± 13.43	18.67 ± 12.13	16.53 ± 13.62	−1.208	0.227
Lifetime suicide attempts	96 (17.5)	16 (24.2)	80 (16.6)	2.373	0.123
Number of suicide attempts	1.41 ± 0.75	1.76 ± 1.09	1.34 ± 0.64	−2.184	0.031
Non suicidal self-harm	47 (8.6)	14 (21.2)	33 (6.8)	15.338	<0.001
Length of hospitalisation (days)	10.56 ± 10.11	13.65 ± 12.40	9.89 ± 10.15	−2.744	0.006
Involuntary admission	158 (28.8)	17 (25.8)	141 (29.2)	0.334	0.563
Physical restraint	71 (12.9)	8 (12.1)	63 (13.0)	0.044	0.834
Psychiatric comorbidity	272 (49.5)	47 (71.2)	225 (46.6)	14.089	<0.001
Medical comorbidity	248 (45.2)	33 (50.0)	215 (44.5)	0.706	0.401
Family history of psychiatric disorders	227 (41.3)	31 (47.0)	196 (40.6)	0.978	0.323

**Table 3:** Presence of illicit substances in the entire sample and in the frequent service users (FSUs) and non-FSUs subgroups.

N (%)	Total Sample (N = 549)	FSUs (N = 66)	Non-FSUs (N = 483)	Chi^2^/ *t*-Test	*p*
At least one substance	235 (42.8)	42 (63.6)	193 (40.0)	13.297	<0.001
Alcohol	159 (29.0)	27 (40.9)	132 (27.3)	5.205	0.023
Cannabinoid	104 (18.9)	30 (45.5)	74 (15.3)	34.338	<0.001
Cocaine	72 (13.1)	22 (33.3)	50 (10.4)	26.913	<0.001
Amphetamines	9 (1.6)	3 (4.5)	6 (1.2)	3.929	0.047
Heroin	41 (7.5)	13 (19.7)	28 (5.8)	16.234	<0.001
Novel substances	9 (1.6)	2 (3.0)	7 (1.4)	0.900	0.343

**Table 4:** Pharmacological treatment of the entire sample and of the frequent service users (FSUs) and non-FSUs subgroups.

N (%) or Mean ± SD	Total Sample (N = 549)	FSUs (N = 66)	Non-FSUs (N = 483)	Chi^2^/ *t*-Test	*p*
Number of medications	3.15 ± 1.47	3.74 ± 1.37	3.07 ± 1.47	−3.486	0.001
Antipsychotics	431 (78.5)	55 (83.3)	376 (77.8)	1.0369	0.309
Oral	405 (73.8)	52 (78.8)	353 (73.1)	0.976	0.323
Long-acting injection	61 (11.1)	9 (13.6)	52 (10.8)	0.484	0.486
Antidepressants	194 (35.3)	26 (39.4)	168 (34.8)	0.540	0.462
Mood stabilizers	291 (53.0)	35 (53.0)	256 (53.0)	0.000	0.997
Benzodiazepines	371 (67.6)	54 (81.8)	317 (65.6)	6.944	0.008
Others	89 (16.2)	21 (31.8)	68 (14.1)	13.452	<0.001

**Table 5:** Logistic regression analysis to explore the potential characteristics associated with the FSU phenomenon.

	B	S.E.	Wald	*p*	Exp(B)	95% CI for EXP
Current Age	0.015	0.013	1.334	0.248	1.015	0.990–1.041
Single status	−0.069	0.424	0.026	0.871	0.934	0.407–2.142
Male gender	−0.600	0.346	3.001	0.083	0.549	0.279–1.082
Personality disorder	0.762	0.397	3.971	0.050	2.141	1.004–4.667
Substance use disorder	0.217	0.540	0.161	0.688	1.242	0.431–3.582
Age at onset	−0.053	0.019	7.641	0.006	0.949	0.914–0.985
Length of hospitalisation	0.030	0.013	5.442	0.020	1.030	1.005–1.057
Non suicidal self-injuries	0.612	0.476	1.655	0.198	1.844	0.726–4.687
Psychiatric comorbidity	0.061	0.374	0.027	0.870	1.063	0.511–2.212
Presence of illicit drugs	0.007	0.501	0.000	0.989	1.007	0.377–2.689
Alcohol	−0.089	0.423	0.044	0.834	0.915	0.399–2.097
Cannabinoid	0.953	0.446	4.559	0.033	2.593	1.081–6.219
Cocaine	0.156	0.434	0.129	0.720	1.168	0.499–2.733
Benzodiazepine therapy	0.614	0.374	2.689	0.101	1.848	0.887–3.848
Other therapy	0.681	0.359	3.594	0.058	1.977	0.977–3.998
Discharge in public residence	0.399	0.357	1.252	0.263	1.490	0.741–2.998
Discharge against medical advice	1.026	0.477	4.623	0.032	2.791	1.095–7.112
Escape attempt from ward	2.443	0.770	10.076	0.002	11.509	2.546–52.024
Constant	−2.781	0.882	9.938	0.002	0.062	

## Data Availability

The data presented in this study are available on request from the corresponding author. The data are not publicly available due to privacy/ethical restrictions.

## Supplementary Materials

The following are available online at https://www.mdpi.com/article/10.3390/diagnostics13030430/s1, Table S1a: logistic regression analysis considering only sociodemographic characteristics associated with FSUs; Table S1b: logistic regression analysis considering sociodemographic and clinical characteristics associated with FSUs.
