# Supplementary material for: Sociodemographic and Clinical Correlates Associated with the Frequent Service Users in an Italian Psychiatric Emergency Department
Source: Diagnostics (Basel). 2023 Jan 25;13(3):430. doi: 10.3390/diagnostics13030430 (PMC9914622; doi:10.3390/diagnostics13030430)
Supplement: Supplementary file 1 [file diagnostics-13-00430-s001.zip › diagnostics-2060010-supplementary.pdf]

Table S1a: logistic regression analysis considering only sociodemographic characteristics associated with FSUs

**Model 1**	B	S.E.	Wald	p	Exp(B)	95% CI for EXP
Current Age	-0.025	0.010	6.689	0.010	0.975	0.956-0.994
Single status	0.169	0.370	.207	0.649	1.184	.573-2.445
Male gender	-0.269	.277	.943	0.332	0.764	0.444-1.316
Constant	-0.899	0.595	2.283	0.131	0.407	

R^2 Nagelkerke=0.041

Table S1b: logistic regression analysis considering sociodemographic and clinical characteristics associated with FSUs

**Model 2**	B	S.E.	Wald	p	Exp(B)	95% CI for EXP
Current Age	0.012	0.013	0.909	0.340	1.012	0.987-1.037
Single status	-0.014	0.407	0.001	0.974	0.987	.444-2.191
Male gender	-0.408	0.327	1.555	0.212	0.665	0.350-1.263
Personality disorder	0.766	0.390	3.865	0.049	2.152	1.002-4.620
Substance use disorder	0.349	0.519	0.454	0.500	1.418	0.513-3.921
Age at onset	-0.050	0.018	7.631	0.006	0.951	0.918-0.986
Length of hospitalisation	0.030	0.012	5.970	0.015	1.031	1.006-1.056
Non suicidal self-injuries	0.567	0.465	1.486	0.223	1.763	0.709-4.385
Psychiatric comorbidity	0.086	0.360	0.058	0.810	1.090	0.538-2.207
Presence of illicit drugs	0.067	0.487	0.019	0.890	1.069	0.412-2.778
Alcohol	-0.182	0.400	0.208	0.648	0.833	0.381-1.824
Cannabinoid	0.994	0.437	5.187	0.023	2.703	1.149-6.362
Cocaine	0.387	0.402	0.923	0.337	1.472	0.669-3.239
Benzodiazepine therapy	0.548	0.362	2.286	0.131	1.730	0.850-3.520
Other therapy	0.524	0.345	2.299	0.129	1.688	0.858-3.322
Constant	-2.557	0.858	8.890	0.003	0.078	

R^2 Nagelkerke=0.224
